# Exhaled Carbon Monoxide Levels in Forty Resistant to Cessation Male Smokers after Six Months of Full Switch to Electronic Cigarettes (e-Cigs) or to A Tobacco Heating Systems (THS)

**DOI:** 10.3390/ijerph16203916

**Published:** 2019-10-15

**Authors:** Fabio Beatrice, Giuseppina Massaro

**Affiliations:** 1 ENT Department and No Smoke Center, St. John Hospital (Head: Beatrice F.), ASL City of Turin, 10154 Turin, Italy; 2 Certified Coach—Free Researcher, 10124 Turin, Italy

**Keywords:** tobacco harm reduction, exhaled carbon monoxide, e-Cig, THS

## Abstract

Cigarette smoke releases several toxic chemicals and carcinogens including carbon monoxide (CO). This study examined the levels of exhaled CO in smokers switching to electronic cigarettes (e-Cigs) or a tobacco heating system (THS) and their level of compliance six months after switching. On the basis of their own preferences, 40 male smokers unwilling or unable to stop smoking were switched to e-Cigs or THSs for six months (20 subjects in each group). Nicotine addiction and levels of carbon monoxide in the exhaled breath (eCO) were measured at baseline (the latter also at six months). The Shapiro Wilk test, graphical methods, Student T test or Mann–Whitney test were used to assess the normal distribution of variables and differences between the two groups after six months. The two groups showed no difference at baseline, but a significant higher addiction score in smokers choosing THS. E-Cig and THS showed significant reduced levels of eCO (both %COHb and COppm) after six months, which were within the range of non-smoker status. Reduced levels of %COHb did not significantly differ between the two groups, whilst the THS group had a significantly lower reduction in levels of COppm vs the e-Cig group (*p* < 0.05). Both e-Cigs and THSs are capable of significantly reducing eCO at least in the medium term, hence constituting a viable tobacco harm reduction approach in smokers who are unwilling or unable to stop smoking.

## 1. Introduction

Tobacco use kills more than seven million people each year [1]. Due to combustion, cigarettes release not just nicotine but several toxic chemicals and carcinogens including carbon monoxide (CO) [2].

In spite of all efforts by experts and the use of nicotine replacement therapies, smokers still find quitting a difficult goal to meet, because of nicotine addiction.

Hence the need for developing and implementing a reduced risk approach through products that do not require combustion to deliver nicotine and so are less likely to release many harmful and potentially harmful chemical constituents (HPHCs) including CO [3].

Currently there are two kinds of smoking reduced risk products: Electronic cigarettes (e-Cig) and tobacco heating systems (THSs), or heat-not-burn product.

E-Cigs consist of a lithium battery, electronic components, an atomizer and a cartridge that holds a liquid solution composed of water, propylene glycol, flavorings and a tobacco-free nicotine solution.

The THS used in this study consists of a tobacco stick (with processed tobacco made from tobacco powder), a holder (which heats the tobacco by means of an electronically controlled heating blade) and a charger that is used to recharge the holder after each use. THS releases nicotine and other volatile compounds by heating the tobacco rod at temperatures not exceeding 350 °C. At these temperatures, tobacco is just heated and, as a consequence, far fewer chemical toxicants (including CO) are formed [4,5].

Recently, Public Health in England has strongly recommended the use of e-Cigs in inveterate smokers, aiming to reduce toxicity in those ones who are unable or do not want to quit [6].

However, the wide variability of e-Cigs on the market is particularly troublesome: Smokers using open systems can modify the level of nicotine or other products whilst those using pre-filled cartridges cannot, and physicians still need to fully understand the advantages and limits of combustion-free smoking products.

Within the recommended approaches for smoking cessation, according to current guidelines [7,8], in case of non-willingness to or inadmissibility of cessation, we aimed the current study at measuring the exposure levels to the combustion marker carbon monoxide, in the exhaled breath (eCO), of subjects fully switching either to e-Cigs or THSs, and at assessing their compliance after six months, aiming to assess the effectiveness of such systems in reducing the exposure to chemicals produced by combustion, which are considered the most important harmful component of smoking, without any purpose of modifying smokers' addiction to nicotine.

Our study was limited to male smokers in order to avoid any potential bias linked to gender differences in smoking cessation. Actually, a comprehensive review of published data from different studies has shown that women have more difficulty maintaining long-term abstinence than men [9]. 

Smokers will be able to make better choices if they are guided by findings of independent studies focused on the relative reduction in exposure risk after switching to innovative combustion-free devices.

## 2. Materials and Methods

A total of 40 smokers (all males; mean age 49.8 years), smoking 21.7 conventional cigarettes per day for 31 years on average, were enrolled in this pilot observational study.

All of them were either unwilling or unable to stop smoking and requesting a switch to reduced risk products.

Prior to enrollment, the study was approved by the Ethics Committee of the ASL City of Turin (Comitato Etico Interaziendale A.O.U. Città della Salute e della Scienza di Torino—A.O. Ordine Mauriziano di Torino), ethical approval code 0058617.

The smokers were offered a switch, based on individual preference, to either a low potential e-Cig (disposable, pre-filled cartridge, low–medium supply power, nicotine 18mg/ml) or a tobacco heating system (THS) 2.2 (sticks with mean nicotine content of 0.50 mg per stick.

Each smoker, before choosing the type of device, was informed of the essential technical features of the two products and provided with 30 min medically assisted face-to-face training on how to correctly use both devices (Jacobacci & Partners, trademark number 013388186) [10].

A full switch within 15 days was requested to each enrolled subject.

Subjects were consecutively enrolled in the two groups (e-Cig and THS) according to their individual preferences until reaching a maximum of 20 smokers per group. 

The remaining group was then completed, enrolling only those who preferred the other device until reaching 20 subjects in the second group.

All 40 smokers were assessed, before switching, for nicotine addiction, using the Fagerstrom test and eCO measurements.

The Fagerstrom test was designed to provide an ordinal measure of nicotine dependence related to cigarette smoking. It contains six items that evaluate the quantity of cigarette consumption, compulsion to use and dependence. In scoring the Fagerstrom test for nicotine dependence, yes/no items are scored from 0 to 1 and multiple-choice items are scored from 0 to 3. The items are summed to yield a total score of 0–10. The higher the total Fagerstrom score, the more intense is the patient’s physical dependence on nicotine [11].

eCO was used as a marker of combustion products—obtained from a single expiratory breath using a hand-held eCO meter (MicroMedical micro CO monitor, SensorMedics Italia srl, Milan, Italy), according to the manufacturer’s recommendations [10]. eCO levels are expressed in ppm (1–6 = non-smoker; 7–10 = light smoker; 11–20 = smoker; >20 = heavy smoker) and %COHb (0.16–0.96 = non-smoker; 1.12–1.60 light smoker; 1.76–3.20 = smoker; >3.20 heavy smoker).

The Shapiro Wilk test and graphical methods were applied to assess the normal distribution of variables. 

The Wilcoxon test for paired data was applied to assess the variance between the before and after switch separately in the two groups. Even if, in this pilot study, the limited sample size would not allow for a formal statistical comparison, the Mann–Whitney U test was applied to assess any difference between the two groups. Analysis was performed with software Stata 14.1 with a *p* value < 0.05 as statistically significant.

## 3. Results

At baseline, the two groups, of note without any heavy smoker, did not differ as to age, number of cigarettes, eCO (even when separately assessed for light smokers and smokers—data not shown), whilst smokers choosing to switch to THSs had an higher mean addiction score (Fagerstrom test) than those switching to e-Cigs (*p* = 0.001) (Table 1).

Nineteen smokers offered the switch declined to use both systems and were therefore not included in the study. All smokers entering the study managed to completely switch within 15 days in both groups. No subject dropped out the study in either group.

Six months after switching, both groups showed a significant reduction of eCO versus baseline (e-Cig = *p* < 0.001; THS = *p* < 0.001) and all subjects showed levels of eCO within the range of non-smoker status, demonstrating a full adherence to the reduced risk products (Table 2, Figures S1 and S2).

Even if the aim of the study was not to make a formal comparison between the two groups in terms of eCO reduction (Figures S3 and S4), due to the limited sample size of this pilot study, the Mann–Whitney U test showed no significant difference as to %COHb levels after six months in the two groups (*p* = 0.37886), whilst the THS group had a significant lower reduction in levels of COppm vs the e-Cig group at six months (*p* < 0.05).

## 4. Discussion

This is the first independent study investigating eCO levels in subjects fully switching, in 15 days’ time, to different devices for six months. Forty subjects (either light smokers or smokers with no heavy smoker at baseline) were offered to switch according to their preference to either e-Cigs or THSs (20 subjects each group). 

Of note, all smokers entering the study managed to completely switch within 15 days in both groups. This can be explained by their high motivation as a consequence of the specific counselling and training they were provided with and by the subsequent individual choice of the system which they perceived as more appropriate.

Before switching, the only difference of note, between the two groups, was a significantly higher Fagerstrom score in smokers choosing THS. Even if it cannot be excluded that such a high score happened just by chance, this suggests that smokers with greater nicotine dependence find THS more appealing than e-Cigs. 

Our findings after six months of switching concur with those from previous short and long-term studies on e-Cigs and THSs [12,13,14].

No subject dropped out the study in either group, which is impressive but not surprising when considering the kind of counselling they received, their high motivation, that the choice was made on the basis of their own preference, the limited sample size in each group and finally the study duration of just six months.

We showed that switching to either e-Cigs or THSs significantly reduces eCO levels in the exhaled breath from smokers after six months, with levels of eCO being within the range of those ranked as non-smokers. 

At six months, eCO levels did not differ among groups in terms of %COHb but subjects switching to THSs showed a significantly lower reduction as to eCO ppm values vs those one switching to e-Cigs (*p* < 0.05).

Formally comparing the results at six months between the two groups was not the aim of the study and we did not collect data that could help in interpreting the difference in eCO ppm. Even if there could be several hypothetical reasons (e.g., THS subjects showed a higher nicotine addiction, on the basis of the different Fagerstrom score, which could have led to a higher heets consumption vs e-Cig pre-filled cartridges; THS subjects could have had smoker partners at home or an undeclared dual use) they still remain merely conjectural and we can just conclude this is a consequence of the limited sample size of this pilot study and that confirmation would need an additional study for a formal comparison with a proper sample. 

When interpreting the study findings, particularly the high level of adherence to both e-Cigs and THSs in extremely motivated former cigarette smokers, caution should be applied due to the observational nature and to the limited sample size of the study. However, the power analysis of the collected data indicated that our study was adequately powered to detect differences between before and after the switch in each separate group (e-Cig and THS).

As to the wider implications of this study, it seems that non-combustible nicotine sources reduce chronic CO exposure in smokers who do not want to quit nicotine intake. Our study also suggests that respecting smokers’ preferences in choosing a specific reduced risk product can be important in assuring full compliance to the switch, at least in the medium term. This is in line with recent findings by the American Cancer Society, showing that the introduction of THS matches coincided with a decline in cigarette sales in Japan [15].

Health Professionals should consider all the options available to a smoking patient for helping them quit. In cases where that is not possible, they can at least induce smokers to switch to combustion-free products (maybe preferring THSs for smokers with higher addiction scores), accepting the limited residual risk as a trade-off for higher likelihood of success for tobacco harm reduction.

## 5. Conclusions

The findings from this study provide new evidence to health care professionals and smokers in order to help them find the best individual choice among tobacco harm reduction approaches whenever the main goal of smoking cessation cannot be met. Both e-Cigs and THSs are viable options, significantly reducing the levels of combustion markers in exhaled breath, warranting a high level of compliance at least at six months after switching and could be chosen on the basis of the extent of addiction to nicotine.

## Figures and Tables

**Table 1:** Baseline data (Q1 = quartile 1; Q3 = quartile 3).

Baseline	e-Cig (*n* = 20)	THS (*n* = 20)	*p* Value
No. of smokers screened to fill the group	62	53	
Mean age (SD)	50.5 (7.0)	49.15 (7.0)	0.546
Median cigarette/day (Q1–Q3)	20 (20-20)	20 (20–21.59	0.333
Mean Fagerstrom score (SD)	4.6 (1.6)	6.7 (1.3)	0.001
eCO			
Median %COHb (Q1–Q3)	1.92 (1.6–2.8)	1.92 (1.6–2.5)	0.849
Median ppm (Q1–Q3)	12 (10–17.5)	12 (10–15.8)	0.849

**Table 2:** eCO data six months after switching and change vs baseline (Q1 = quartile 1; Q3 = quartile 3).

	After 6 Months	After 6 Months	Change vs. Baseline	Change vs. Baseline	*p*
	Median	Q1; Q3	Median	Q1; Q3	
**%CoHb**					
e-Cig	0.32	0.16; 0.44	−1.6	−2.24; −1.28	*p* < 0.001
THS	0.48	0.48; 0.64	−1.28	−1.92; −1.12	*p* < 0.001
**ppm**					
e-Cig	2	1; 2.75	−10	−10; −8	*p* < 0.001
THS	3	3; 4	−8	−12; −7	*p* < 0.001

## Supplementary Materials

The following are available online at https://www.mdpi.com/1660-4601/16/20/3916/s1, Figure S1: %COHb before and after switch; Figure S2: CO ppm before and after switch; Figure S3: Difference %COHb before and after switch in the two groups; Figure S4: Difference CO ppm before and after switch in the two groups.
