# Supplementary material for: Exhaled Carbon Monoxide Levels in Forty Resistant to Cessation Male Smokers after Six Months of Full Switch to Electronic Cigarettes (e-Cigs) or to A Tobacco Heating Systems (THS)
Source: Int J Environ Res Public Health. 2019 Oct 15;16(20):3916. doi: 10.3390/ijerph16203916 (PMC6843400; doi:10.3390/ijerph16203916)
Supplement: Supplementary file 1 [file ijerph-16-03916-s001.pdf]

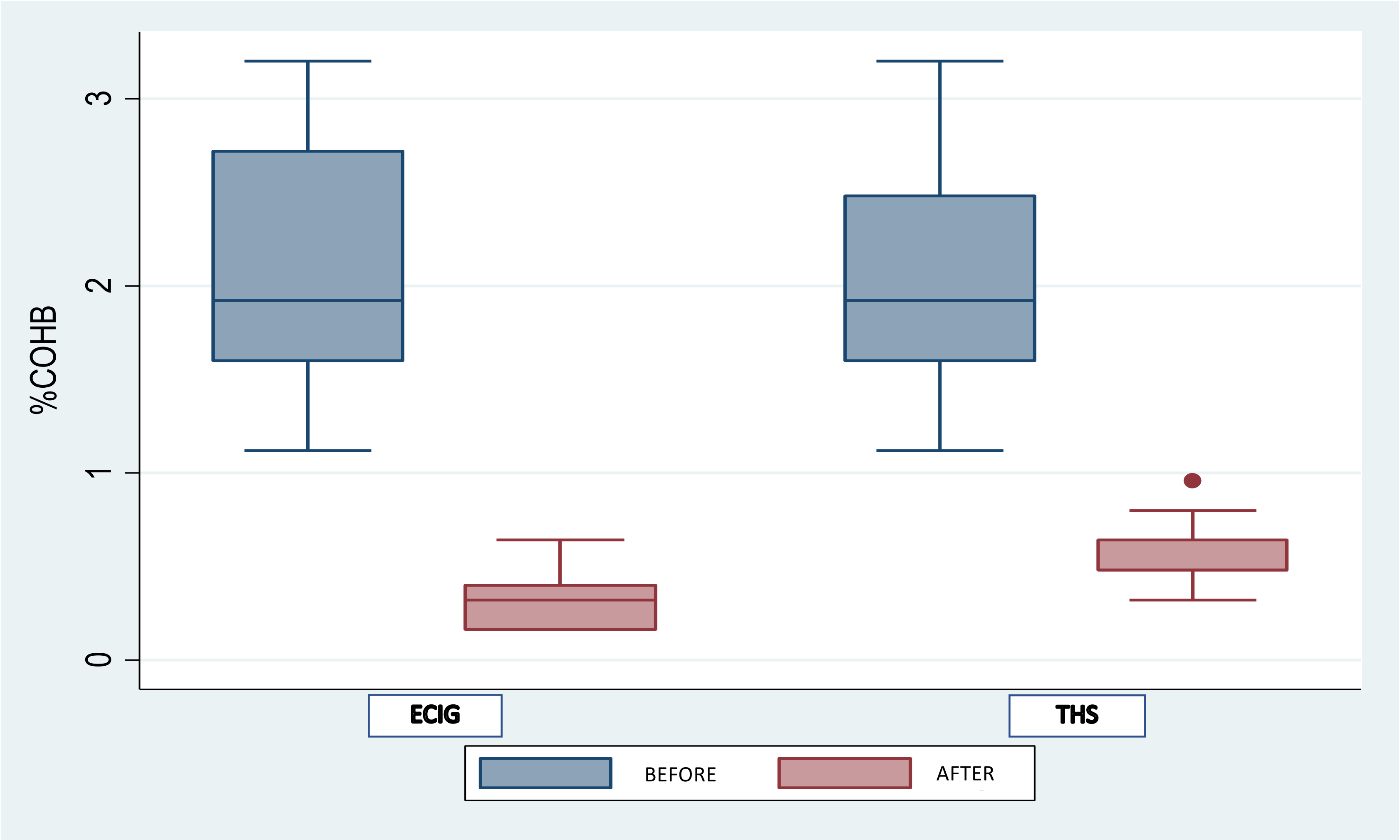


Figure 1: %COHb before and after switch

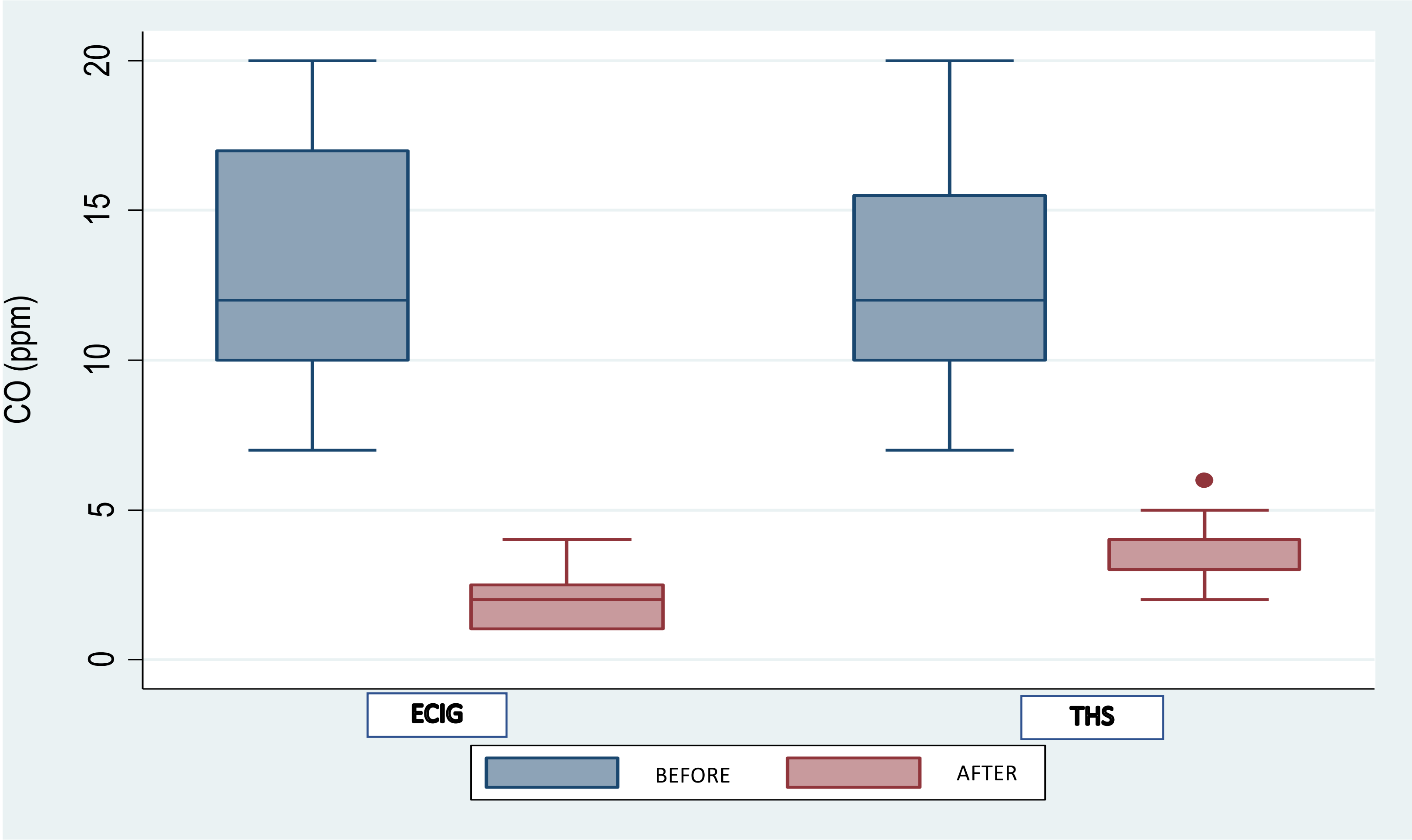


Figure 2: CO ppm before and after switch

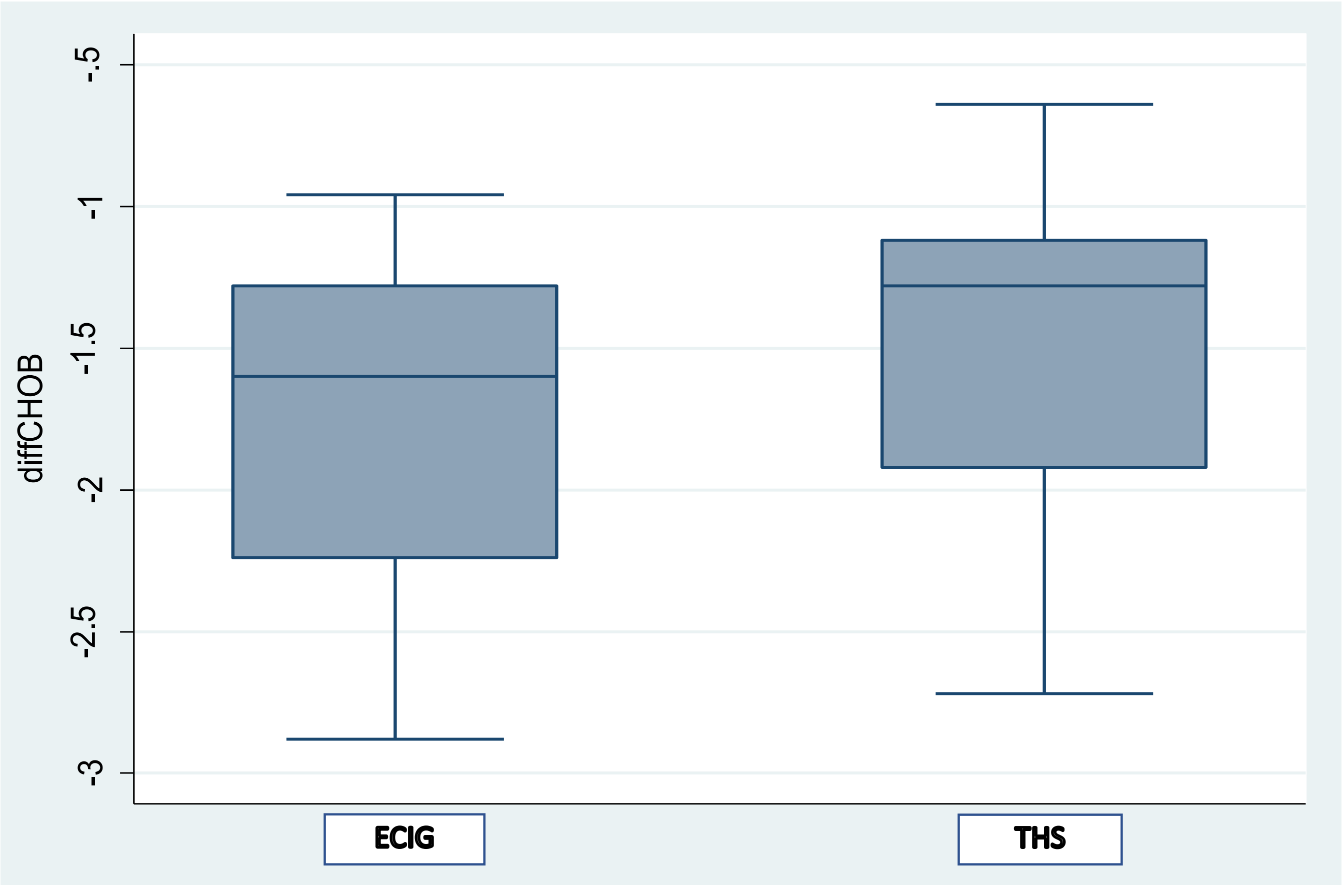


Figure 3: Difference %COHb before and after switch in the 2 groups

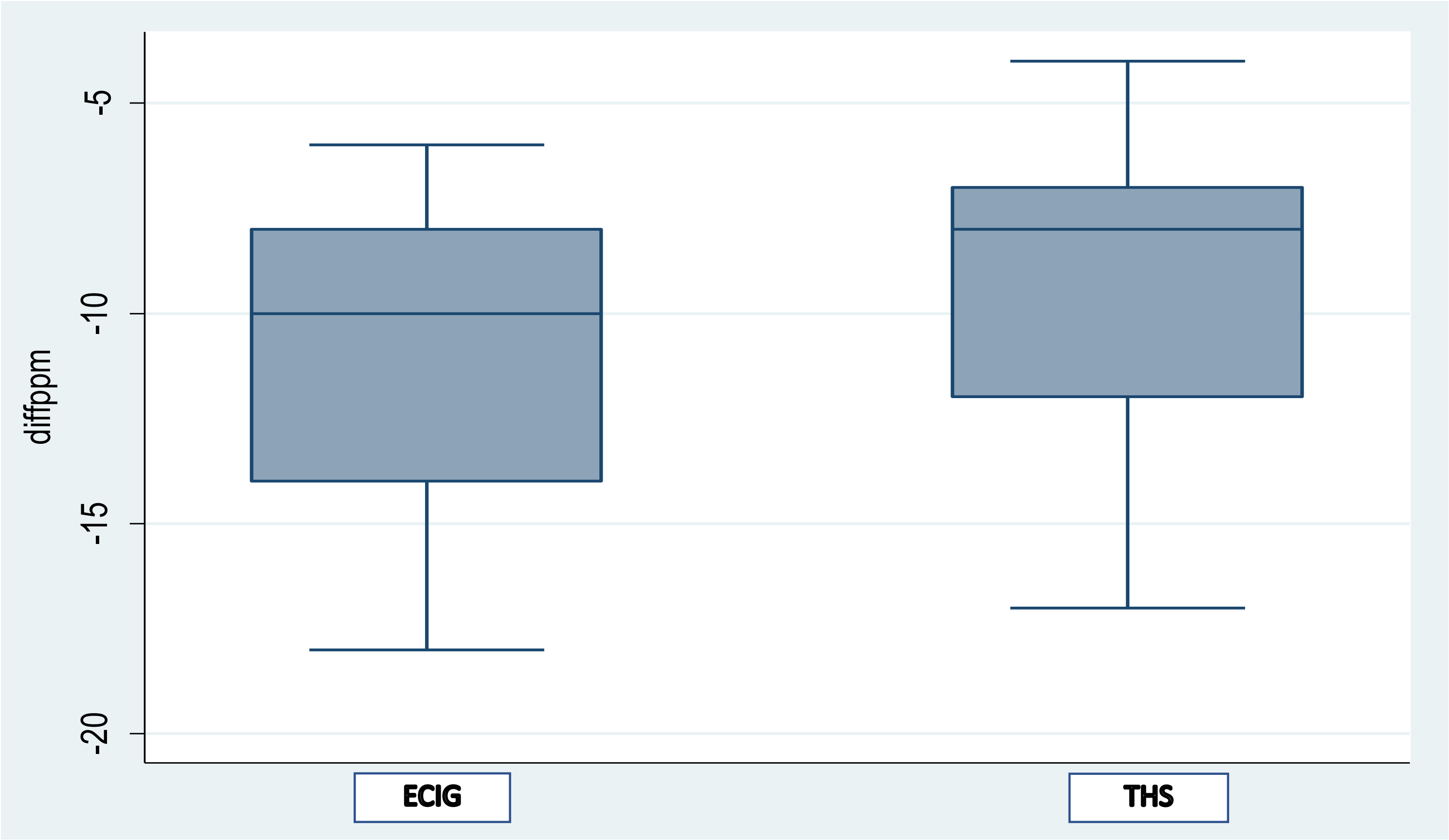


Figure 4: Difference CO ppm before and after switch in the 2 groups
